# Characteristics of lymph node (No.5 and No.6) metastasis and significance of lymph node dissection in Siewert type II esophagogastric junction adenocarcinoma (AEG): No.5 and No.6 lymph node metastases of AEG and clearance

**DOI:** 10.1097/MD.0000000000027106

**Published:** 2021-09-03

**Authors:** Xu Zhang, Xiao-dong He, You-cheng Zhang, Ke-hu Yang, Jin-hui Tian, Yao-long Chen

**Affiliations:** a General Department 2, Second Hospital, Lanzhou University, 82 Cuiyingmen Lanzhou, Gansu, China; b Evidence-Based Medicine Center, School of Basic Medical Sciences, Lanzhou University, Lanzhou, Gansu, China; c Key Laboratory of Evidence-Based Medicine and Knowledge Translation of Gansu Province, Lanzhou, Gansu, China.

**Keywords:** esophagogastric junction adenocarcinoma, No.5 lymph node, No.6 lymph node, Siewert type II

## Abstract

**Background:**

To analyze the characteristics, related risk factors, and prognosis of lymph node metastasis (Number [No.] 5 and No.6) in the group of adenocarcinoma of esophagogastric junction (AEG).

**Methods:**

The patients with Siewert II AEG who underwent total gastrectomy and D2 lymph node dissection from September 2015 to December 2018 in Lanzhou University Second Hospital were enrolled in this study. The pathological features of the postoperative specimens were analyzed (sex, age, maximum diameter, location, depth of invasion, degree of differentiation, neurological and vascular invasion, etc), and the lymph node metastasis rate of No.5, No.6 groups were calculated. The analysis was performed by IBM SPSS statistical software. The risk factors associated with lymph node metastasis in No.5 and No.6 groups were analyzed. Survival analysis was performed by Kaplan-M method, and survival rate was estimated, Log-rank test was used for comparison, and the difference was statistically significant at *P* < .05.

**Results:**

There were 142 cases of Siewert type II AEG with the positive rate of No.5 lymph nodes being 10.81% (8/74), and the positive rate of No.6 lymph nodes was 8.33% (11/132). No.5 and No.6 lymph nodes metastasis were not associated with gender, age, tumor maximum diameter, location (cardiac left/cardiac right) (*P* > .05), and were associated with invasion depth, differentiation degree, nerve and vascular invasion (*P* < .05). In the No.5 lymph node-positive group, the 3-year Overall Survival (OS) was 25.0%, and the No.5 lymph node-negative group had a 5-year OS of 57.8%, which was statistically different (*P* < .05). The 3-year OS was 18.2% in No.6 node-positive group and 53.8% in No.6 node-negative group, and the difference was statistically significant (*P* < .05).

**Conclusion:**

For Siewert type II AEG, the lymph node metastasis rate was higher in No.5 and No.6 groups when the tumor invaded all layers of gastric wall and was poorly differentiated complicated with vascular nerve invasion, and the lymph node metastasis rate was lower at 3 years, which may be more appropriate for total gastrectomy +D2 lymph node dissection.

## 1 Introduction

In recent years, the incidence of adenocarcinoma of esophagogastric junction (AEG) has increased in all regions of the world. However, with the advancement of science and technology, the mortality rate has not decreased and the prognosis is worse than that of lower gastric tumors, thus causing widespread concern among scholars.^[1–3]^ Due to the specific anatomical location, surgical treatment is performed by both thoracic and general surgeons, respectively, and the surgical approach and extent of lymph node dissection have been controversial.^[4,5]^ The surgical route is closely related to the location and size of the tumor. Currently, the Siewert staging is commonly used for AEG.^[6]^ For Siewert type I and Siewert type III, there is less controversy, but the surgical approach extent of esophageal resection and extent of lymph node dissection for Siewert type II AEG are still controversial.^[5–7]^ The controversy has focused on the radicality of surgery and the quality of postoperative survival in Siewert II AEG, with the rate of lymph node metastasis in the Number (No.) 5 and No.6 groups playing an important role in the choice of surgical approach. In this paper, we analyze the No.5 and No.6 lymph node metastases in Siewert II AEG, analyze the associated risk factors, and investigate the impact of lymph node metastases in the No.5 and No.6 groups on the 3-year survival rate, in order to provide a reference for the surgical approach to Siewert II AEG.

## 2 Materials and methods

### 2.1 Inclusion criteria

Data were collected on Siewert II AEG patients from September 2015 to December 2018 at our hospital, of which 142 cases met the inclusion criteria. Inclusion criteria: ① surgery: all surgeons received uniform training, the surgical approach was transabdominal esophageal fissure pathway, and total gastrectomy +D2 lymph node dissection was completed (all met the criteria for D2 radical treatment of gastric cancer); ② postoperative pathology: adenocarcinoma with the center of the tumor located 1 cm above the dentate line and 2 cm below the dentate line^[6]^; ③ no radiotherapy treatment before surgery; ④ no distant metastasis before surgery; ⑤ postoperative pathology with valid perigastric group 1 to 6 lymph nodes were recorded; ⑥ the number of postoperative lymph nodes was greater than 16; ⑦ the postoperative pathology of group No.5 or No.6 lymph nodes were clearly recorded; ⑧ the follow-up data were well documented and the postoperative adjuvant treatment factors were consistent.

### 2.2 General information

This research was obtained using the American Joint Committee on Cancer/International Union Against Cancer 8th edition of the AEG Tumor Node Metastasis staging scheme.^[8]^ Baseline information of patients was as follows: a total of 142 cases were included in the study, 117 males and 25 females, aged (66.0 ± 7.8) years. The maximum tumor diameter (d) was ≤3 cm in 26 cases, 3 cm < d ≤ 5 cm in 61 cases and d >5 cm in 55 cases. Tumor T-stage: T1 – 236 cases, T3 – 4106 cases; Tumour N-stage: N0 – 150 cases, N2 – 392 cases. Tumor Node Metastasis stage: 12 cases in stage I, 32 cases in stage II, 58 cases in stage III, 40 cases in stage IV. Eight cases in group No.5 had positive lymph nodes, 66 cases in group No.5 had negative lymph nodes, 68 cases in group No.5 had no lymph nodes in postoperative pathology; Group No.6: There were 11 positive lymph nodes in group No.6, 121 negative lymph nodes in group No.6, and no lymph nodes in group No.6 in 10 postoperative pathologies. There were 95 cases of nerve invasion and 39 cases of non-invasion, and 67 cases of vascular invasion and 67 cases of non-invasion (8 of the 142 postoperative pathologies were not marked with nerve or vascular invasion). The center of the tumor was located on the right side of the cardia in 17 cases and on the left side of the cardia in 125 cases.

### 2.3 Research methods

The risk factors associated with lymph node metastasis in the No.5 and No.6 groups were analyzed, and the 3-year survival rates of the No.5 and No.6 groups with positive and negative lymph nodes were statistically analyzed. IBM SPSS Statistics for Windows, version 20.0. (Armonk, NY) statistical software was applied for analysis. *t* test was used for the mean and χ2 test for the rate, and *P* < .05 was considered a statistically significant difference. The Kaplan-M method was used for survival analysis and survival rates were calculated. Log-rank test was used for comparison and *P* < .05 was considered statistically significant.

## 3 Results

### 3.1 Lymph node detection

One hundred forty two cases of Siewert II AEG in this group, lymph node detection: group No.1 (9.84 ± 5.35), group No.2 (4.32 ± 2.36), group No.3 (8.54 ± 5.23), group No.4 (5.01 ± 3.87), group No.5 (1.35 ± 1.98), group No.6 (3.63 ± 2.83). A total of 4544 lymph nodes were detected in the surgical specimens, each (33.37 ± 12.54) 74 lymph nodes were detected in the No.5 group, ranging from 1 to 15 nodes each, and a total of 191 lymph nodes were cleared; 8 positive lymph nodes were present in the No.5 group, ranging from 1 to 14 nodes each, and a total of 28 positive lymph nodes were cleared. One hundred thirty two lymph nodes were detected in the No.6 group, ranging from 1 to 18 nodes each, and a total of 516 lymph nodes were cleared. A total of 516 lymph nodes were cleared, of which 11 cases in the No.6 group had positive lymph nodes, ranging from 1 to 5 nodes per case, with a total of 28 positive lymph nodes.

### 3.2 The rate of lymph node metastasis

It was 16.67% in the early stage AEG and 79.23% in the progressive stage AEG, 61.97% in group No.1 (88/142), 51.45% in group No.2 (71/138), and 58.45% in group No.3 (83/142), No.4 group had a transfer rate of 13.74% (16/131), No.5 group had a transfer rate of 10.86% (8/74), and No.6 group had a transfer rate of 8.33% (11/132).

### 3.3 Factors associated with lymph node metastasis in groups No.5 and No.6

According to whether the lymph nodes in groups No.5 and No.6 metastasized, the cases with metastasis in the suprapyloric region were classified as positive in group No.5 (8 cases), and the cases without metastasis were classified as negative in group No.5 (66 cases); The cases with metastasis in the subpyloric region were classified as positive in group No.6 (11 cases), and the cases without metastasis were classified as negative in group No.6 (121 cases). The risk factors associated with lymph node metastasis in the No.5 group were: T-stage, degree of differentiation, nerve and vascular infiltration (*P* < .05), see Table 1; the risk factors associated with lymph node metastasis in the No.6 group were: T-stage, degree of differentiation, nerve and vascular infiltration (*P* < .05), see Table 2.

**Table 1:** Analysis of risk factors associated with lymph node metastasis in the No.5 group.

Study factors	No.5 positive group (8 cases)	No.5 negative group (66 cases)	χ2 (t) value	*P* value
Sex			0.000	1.000
Male	6	53		
Female	2	13		
Age (years)	67.1 ± 9.9	66.8 ± 8.0	0.448	.656
Maximum tumor diameter			0.044	.835
≤3 cm	1	15		
>3 cm and ≤5 cm	4	24		
>5 cm	3	27		
Tumor location			0.000	1.000
Cardia left	7	57		
Cardia right	1	9		
Depth of invasion				.048^∗^
T1–2	0	24		
T3–4	8	42		
Degree of tumor differentiation			5.914	.015
Hypofractionated	4	7		
Intermediate-highly differentiated	4	59		
Vascular infiltration				.001^∗^
(+)	8	24		
(−)	0	36		
Nerve infiltrate			5.071	.024
(+)	7	23		
(−)	1	37		

Six cases in group No.5 (−) had postoperative pathology without nerve or vascular invasion, all units are “cases” except age. No. = Number.

∗ Fisher exact probability method was used.

**Table 2:** Analysis of risk factors associated with lymph node metastasis in the No.6 group.

Study factors	No.6 positive group (11 cases)	No.6 negative group (121 cases)	χ2 (t) value	*P* value
Sex			0.000	1.000
Male	9	100		
Female	2	21		
Age (years)	65.3 ± 8.9	66.2 ± 7.8	–0.367	.714
Maximum tumor diameter			2.083	.149
≤3 cm	1	23		
>3 cm and ≤5 cm	3	54		
>5 cm	7	44		
Tumor location			0.026	.872
Cardia left	9	107		
Cardia right	2	14		
Depth of invasion				.037^∗^
T1–2	0	35		
T3–4	11	86		
Degree of tumor differentiation			12.615	.000
Hypodifferentiated	8	24		
Intermediate-highly differentiated	3	97		
Vascular infiltration			4.267	.039
(+)	9	50		
(−)	2	63		
Nerve infiltrate				.033^∗^
(+)	11	78		
(−)	0	35		

Eight cases in the No.6 (−) group had postoperative pathology without nerve or vascular invasion, all units are “cases” except age. No. = Number.

∗ Fisher exact probability method was used.

### 3.4 The follow-up rate

**Figure 1:**
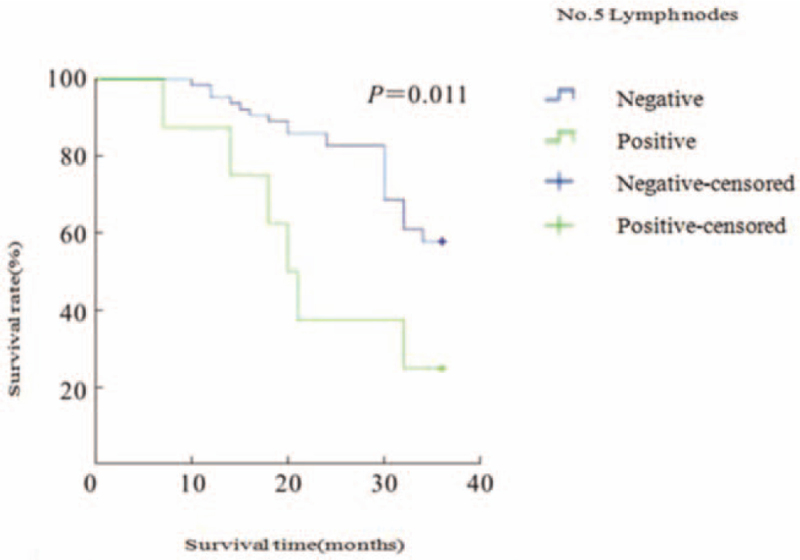
Survival curves for the No.5 lymph node positive group and the negative group. No. = Number.

**Figure 2:**
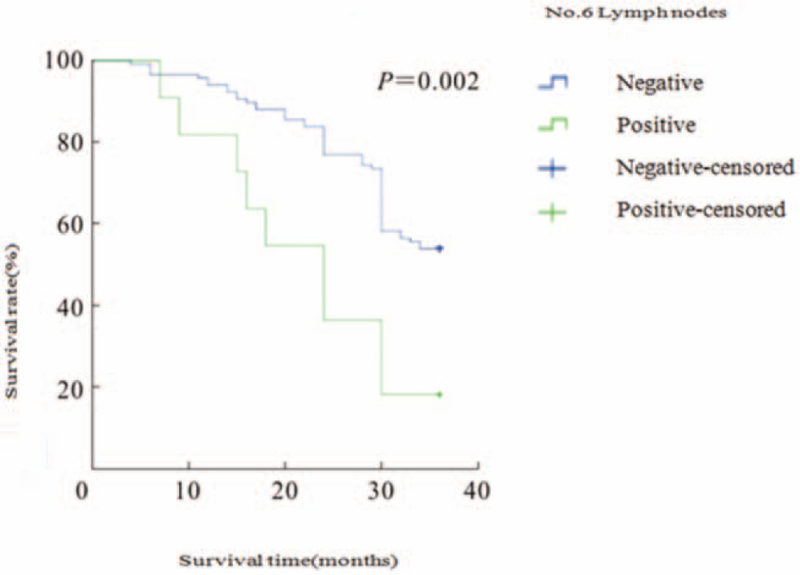
Survival curves for the No.6 lymph node positive group and the negative group. No. = Number.

It was 100% (8/8), 96.9% (64/66) for the No.5 negative group, 100% (11/11) for the No.6 positive group, and 96.7% (117/121) for the No.6 negative group; The 3-year survival rates for the No.5 and No.6 lymph node positive groups and the negative group are shown in Table 3, and the survival curves for the 4 groups are shown in Figures 1 and 2. The curves are shown in Figures 1 and 2.

**Table 3:** Three-year survival rate of lymph node positive and negative groups No.5 and No.6.

Group	Number of cases	3-year survival rate	χ2 value	*P* value
No.5 lymph nodes			6.439	.011
(+)	8	25.0		
(−)	66	57.8		
No.6 lymph nodes			9.875	.002
(+)	11	18.2		
(−)	121	53.8		

No. = Number.

## 4 Discussion

### 4.1 Significance of lymph node dissection in AEG

In this study, the lymph node metastasis rate was 16.67% in patients with early stage AEG and 79.23% in progressive stage AEG. A related study in Japan^[9]^ showed that AEG has a high rate of lymph node metastasis and a poor prognosis compared with non-esophagogastric union gastric adenocarcinoma. A related study in China^[10]^ also reported that AEG had a large primary lesion, a high rate of lymph node metastasis, a late pathological stage, and a poor prognosis compared to adenocarcinoma of the gastric sinus. Therefore, both early-stage AEG and progressive AEG need to be thoroughly cleared of the corresponding lymph nodes to improve the survival rate. The lymph node staging of Siewert I and Siewert III AEG and the extent of lymph node dissection are less debated and there is consensus +D2 lymph node dissection, if cT1N0 and tumor diameter <4 cm, D1+ lymph node dissection is feasible.^[11]^

### 4.2 The influence of the lymph node metastasis rate in groups No.5 and No.6 on the choice of surgical approach for Siewert type II AEG

It is still controversial as the extent of Siewert type II AEG clearance is not clearly defined, especially for lymph node clearance in the pyloric region, which to some extent determines the choice of surgical approach.^[4]^ Goto et al^[9]^ showed that Siewert type II AEG, the lymph node metastasis rate was 2.7% in group No.5 and 0 in group No.6. The benefit of lymph node dissection was small and proximal gastrectomy without routine dissection of the lymph nodes in the pyloric region was recommended. In China, Cao et al^[12]^ showed that the lymph node metastasis rate in Siewert II AEG was 1.4% in the No.5 group and 0.7% in the No.6 group, with a low metastasis rate and negligible benefit of lymph node dissection in the pyloric region, regardless of the T-stage and differentiation of the tumor. However, some scholars in Japan also reported^[13]^ that in Siewert II AEG, when the lower margin of the tumor was greater than 5 cm from the dentate line, the metastasis rate of lymph nodes in the pyloric region could reach 20%, and complete lymph node dissection of groups No.5 and No.6 was required, and total gastrectomy was performed. A similar study was also reported in China,^[14]^ in which the lymph node metastasis rate of Siewert II AEG was 18.82% for group No.5 and 12.82% for No.6, and it was suggested that total gastrectomy is recommended for Siewert II AEG cases, especially for patients with large tumors (>4 cm in diameter) and T-stage 3 to 4. The proximal versus total gastrectomy approach to AEG has been debated, mainly around the radicality of the tumor and the quality of life after surgery. The advantages of proximal gastrectomy over total gastrectomy are less invasive, less technically demanding and a lower risk of postoperative malnutrition and anemia.^[15]^ However, proximal gastrectomy also has its disadvantages, such as reflux esophagitis, anastomotic stricture, and swallowing difficulties.^[12]^ The most fundamental reason why proximal gastrectomy is not used as the standard procedure for Siewert II AEG may still be the ability to completely clear the tumor lymph nodes. Proximal gastrectomy may not be able to effectively and completely clear the No.5 and No.6 group of lymph nodes while preserving the right gastric artery and vein and the right gastroretinal artery and vein.

### 4.3 Lymph node metastasis rate and risk factors in groups No.5 and No.6

In this study, the metastasis rate was 10.86% in the No.5 group and 8.33% in the No.6 group, which was significantly lower compared to the 61.97% metastasis rate in the No.1 group, 51.45% in the No.2 group and 58.45% in the No.3 group, but should not be ignored, which is similar to the results of some studies.^[13,14]^ Risk factors for lymph node metastasis in the No.5 and No.6 groups included T4, hypofractionation, nerve and vascular infiltration. The maximum tumor diameter did not correlate with lymph node metastasis in groups No.5 and No.6 (*P* > .05), which was inconsistent with the results of most studies in China.^[14,16]^ it was considered that this might be related to the heterogeneity and specific biological behavior of the tumor, which grew laterally along the mucosa of the gastric wall without infiltrating longitudinally. At the time of pathological statistics, it was found that in several cases the maximum diameter of the cancer infiltration was greater than 3 cm, while the depth of infiltration was only mucosal, without lymph node metastasis.

### 4.4 Effect of lymphatic metastases on survival in groups No.5 and No.6

In this study, the 3-year survival rate was 25.0% in the No.5 lymph node positive group, 57.8% in the No.5 lymph node negative group, 18.2% in the No.6 lymph node positive group and 53.8% in the No.6 lymph node negative group, with the positive group having a worse survival rate than the negative group. The difference between the 2 groups was statistically significant (*P* < .05). The results suggest that for Siewert II AEG, if there is metastasis in the lymph nodes in the pylorus region, the prognosis is poor and the lymph node dissection should be expanded to completely dissect the pylorus region, and total gastrectomy +D2 lymph node dissection is more appropriate. This study has the following shortcomings: a retrospective, single-center, small-sample study may have a selection bias; only 3-year survival rates were analyzed for follow-up data, which may lead to biased survival rates. In conclusion, we believe that for Siewert II AEG, if the tumor is pre-operatively and intra-operatively found to be T4, hypofractionated, with neurological and vascular invasion, the rate of lymph node metastasis in the pyloric region is higher and total gastrectomy +D2 lymph node dissection is the more appropriate surgical approach.

## Acknowledgments

We are grateful to Mr Xiao-dong He and Mr You-cheng Zhang of the Second Hospital of Lanzhou University for their support in surgery and data management, and to Mr Ke-hu Yang, Mr Jin-hui Tian, and Mr Yao-long Chen of the Evidence-based Centre of the School of Basic Medicine of Lanzhou University for their support in statistical data.

## Author contributions

All authors made substantial contributions to conception and design, acquisition of data, or analysis and interpretation of data; Took part in drafting the article or revising it critically for important intellectual content; Gave final approval of the version to be published and agree to be accountable for all aspects of the work.

**Conceptualization:** Xu Zhang, Xiao-dong He, You-cheng Zhang, Ke-hu Yang, Jin-hui Tian, Yao-long Chen.

**Data curation:** Xu Zhang, Xiao-dong He, You-cheng Zhang, Ke-hu Yang, Jin-hui Tian, Yao-long Chen.

**Formal analysis:** Xu Zhang, You-cheng Zhang, Ke-hu Yang, Jin-hui Tian, Yao-long Chen.

**Investigation:** Xu Zhang, Xiao-dong He, You-cheng Zhang, Ke-hu Yang, Jin-hui Tian, Yao-long Chen.

**Methodology:** Xu Zhang, Xiao-dong He, You-cheng Zhang, Ke-hu Yang, Jin-hui Tian, Yao-long Chen.

**Project administration:** Xu Zhang, Xiao-dong He, You-cheng Zhang.

**Resources:** Xu Zhang, Xiao-dong He, You-cheng Zhang, Yao-long Chen.

**Software:** Xu Zhang, You-cheng Zhang, Ke-hu Yang, Jin-hui Tian, Yao-long Chen.

**Supervision:** Xiao-dong He, You-cheng Zhang, Ke-hu Yang, Jin-hui Tian.

**Validation:** Xu Zhang, Xiao-dong He, You-cheng Zhang, Jin-hui Tian, Yao-long Chen.

**Visualization:** Xu Zhang, Xiao-dong He, You-cheng Zhang, Yao-long Chen.

**Writing – original draft:** Xu Zhang, Xiao-dong He.

**Writing – review & editing:** Xiao-dong He, You-cheng Zhang, Ke-hu Yang, Jin-hui Tian, Yao-long Chen.

## References

[1] Colquhoun A Arnold M Ferlay J Goodman KJ Forman D Soerjomataram I. Global patterns of cardia and non-cardia gastric cancer incidence in 2012. Gut 2015;64:1881–8. 25748648 10.1136/gutjnl-2014-308915

[2] Wang R Chen XZ. High mortality from hepatic, gastric and esophageal cancers in mainland China: 40 years of experience and development. Clin Res Hepatol Gastroenterol 2014;38:751–6. DOI: 10.1016/j.clinre.2014.04.014. 24994519 10.1016/j.clinre.2014.04.014

[3] Liu K Zhang WH Chen XL. Comparison on clinicopathological features and prognosis between esophagogastric junctional adenocarcinoma (Siewert II/III types) and distal gastric adenocarcinoma. Medicine 2015;94:e1386 DOI: 10.1097/md.0000000000001386. 26313779 10.1097/MD.0000000000001386 PMC4602903

[4] Cao F Zhao EH. Current status and controversies in the surgical treatment of adenocarcinoma of the esophagogastric junction. Chin J Pract Surg 2016;36:62–6.

[5] Hatta W Tong D Lee YY Ichihara S Uedo N Gotoda T. Different time trend and management of esophagogastric junction adenocarcinoma in three Asian countries. Dig Endosc 2017;29:18–25. 28425657 10.1111/den.12808

[6] Siewert JR Stein HJ. Classification of adenocarcinoma of the oesophagogastric junction. Br J Surg 1998;85:1457–9. 9823902 10.1046/j.1365-2168.1998.00940.x

[7] Yang W Yuan Y Hu Hao-yuan. Prognosis analysis of Siewert type II adenocarcinoma of the esophagogastric junction by transthoracic versus transabdominal approach. Chin J Gastroint Surg 2019;22:132–42. DOI:. 30799535

[8] Amin MB Edge SB Greene FL. Organization of the AJCC cancer staging manual // AJCC Cancer Staging Manual. 2016;Cham: Springer International Publishing, 31–37.

[9] Goto H Tokunaga M Miki Y. The optimal extent of lymph node dissection for adenocarcinoma of the esophagogastric junction differs between Siewert type II and Siewert type III patients. Gastric Cancer 2015;18:375–81. 10.1007/s10120-014-0364-0 PMC4371819 24658651

[10] Zhu ZY Wang YM Li FK. Comparison of the clinicopathological features and prognosis of adenocarcinoma of the esophagogastric junction and adenocarcinoma of the gastric sinus. Chin J Gastroint Surg 2019;22:149–55. 30799537

[11] Brown LM Devesa SS Chow WH. Incidence of adenocarcinoma of the esophagus among white Americans by sex, stage, and age. J Natl Cancer Inst 2008;100:1184–7. 18695138 10.1093/jnci/djn211 PMC2518165

[12] Cao HH Ooi M Yu Z. Should pyloric lymph nodes be dissected for Siewert type II and III adenocarcinoma of the esophagogastric junctions: experience from a high-volume center in China. J Gastrointest Surg 2019;23:256–63. 30334176 10.1007/s11605-018-3935-2

[13] Mine S Kurokawa Y Takeuchi H. Distribution of involved abdominal lymph nodes is correlated with the distance from the esophagogastric junction to the distal end of the tumor in Siewert type II tumors. Eur J Surg Oncol EJSO 2015;41:1348–53. 26087995 10.1016/j.ejso.2015.05.004

[14] Li M Meng QB Shao YS. Significance of peripyloric lymph node clearance in Siewert type II adenocarcinoma of the esophagogastric junction. Abdom Surg 2018;31:238–40, 244.

[15] Huh YJ Lee HJ Oh SY. Clinical outcome of modified laparoscopy-assisted proximal gastrectomy compared to conventional proximal gastrectomy or total gastrectomy for upperthird early gastric cancer with special references to postoperative reflux esophagitis. J Gastric Cancer 2015;15:191 DOI: 10.5230/jgc.2015.15.3.191. 26468417 10.5230/jgc.2015.15.3.191 PMC4604334

[16] Qian T Zhou SY. Exploration of the pattern of lymph node metastasis in Siewert type II adenocarcinoma of the esophagogastric junction. Shandong Med 2013;53:72–3.

